# Validation of the French version of the Amsterdam preoperative anxiety and information scale (APAIS)

**DOI:** 10.1186/1477-7525-11-166

**Published:** 2013-10-07

**Authors:** Axel Maurice-Szamburski, Anderson Loundou, Xavier Capdevila, Nicolas Bruder, Pascal Auquier

**Affiliations:** 1 Service d’Anesthésie Réanimation Pr BRUDER, Hôpital TIMONE Adulte, 264 rue Saint Pierre, 13385 Marseille Cedex 05, France; 2 Unité d’aide méthodologique, Direction de la Recherche Clinique AP-HM, Marseille, France; 3 Service d’Anesthésie Réanimation, Hôpital Lapeyronie and Inserm U 1046, Montpellier, France; 4 Santé publique, EA3279, Faculté de Médecine, Marseille, France

## Abstract

**Background:**

Most patients are anxious before surgery. The level of preoperative anxiety depends on several factors and merits an objective evaluation. The Amsterdam Preoperative Anxiety and Information Scale (APAIS) is a self-report questionnaire comprising six questions that have been developed and validated to evaluate the preoperative anxiety of patients. This global index assesses three separate areas: anxiety about anaesthesia, anxiety about surgery, and the desire for information. The purpose of this study was to translate the APAIS into French and to evaluate the psychometric properties of the French version of the APAIS.

**Methods:**

The process consisted of two steps. The first step involved the production of a French version of the APAIS that was semantically equivalent to the original version. In the second step, we evaluated the psychometric properties of the French version, including the internal consistency and reliability, the differential item functioning, and the external validity. Participants older than 18, undergoing elective surgery (except obstetric), able to understand and read French, and able to complete a self-report questionnaire were eligible for inclusion in the study. A forward-backward translation was performed. The psychometric evaluation covered three domains: internal validity, external validity, and acceptability. Within 4–48 h after surgery, the patients were asked to complete the “Evaluation du Vécu de l’ANesthésie” questionnaire” (EVAN) questionnaire, which is a validated, multi-dimensional questionnaire that assesses the patient’s experiences in the perioperative period.

**Results:**

A database with 175 patients was created. The principal component factor analysis revealed the same three-dimensional structure as the original scale. The confirmatory factor analysis showed a strong fit with a root mean square error of approximation of 0.069 and a comparative fit index of 1.00. The amount of differential item functioning (DIF) between the subgroups of patients (i.e., based on age, gender, type of anaesthesia or surgery, premedication, ASA physical status, and ambulatory course) was low. The APAIS was strongly correlated with the dimensions of the EVAN. Each dimension had a low proportion of missing values (ranging from 0.6 to 2.9%), which indicates good acceptability of the questionnaire.

**Conclusions:**

The French version of the APAIS is valid and reliable. The availability of this tool enables the evaluation of anxiety in French patients undergoing anaesthesia.

## Introduction

Approximately 60% of patients undergoing surgery are anxious [1]. The addressing of anxiety is a serious concern for the improvement of the patient experience during the perioperative period. Moreover, preoperative anxiety can lead to adverse effects, such as autonomic fluctuation and resistance to anaesthetic induction. These problems justify the widespread prescription of sedative premedication around the world [2,3], but these problems may not necessarily be related to the real level of anxiety experienced by the patients. The level of preoperative anxiety depends on several factors [4-6], and it is difficult to objectively evaluate anxiety. Most of the time, physicians attempt to rate their patients’ anxiety themselves, which leads to variable results [7].

There are several instruments for measuring preoperative anxiety in patients. One of the most used is the Spielberger’s State-Trait Anxiety Inventory (STAI) [8], consisting in two different scale: one for measuring “trait” anxiety, the other for measuring “state” anxiety. But even if the “state” of STAI scale is aimed to assess a situation related anxiety, it takes too much time to be fulfilled to be usable in the perioperative framework.

Beside anxiety, patients need for information is an important aspect that should be assessed because of its weight in the patient global experience of the perioperative period [9]. But despite its importance, there was no instrument forecasted to assess patients need for information, until the Amsterdam Preoperative Anxiety and Information Scale (APAIS).

The Amsterdam Preoperative Anxiety and Information Scale (APAIS) is a self-report questionnaire composed of six questions that were developed and validated to evaluate a patient’s preoperative anxiety. This global index includes three separate areas: anxiety about anaesthesia, anxiety about surgery, and the desire for information. The items are rated on a five-point Likert scale from “not at all” to “extremely” [10]. The APAIS has been validated in surgical patients, whereas the STAI scale was validated in the general population [8]. Thus, using only six items, the APAIS may become the standard for the evaluation of patient anxiety in the perioperative period if it is available and validated in all languages [11]. Moreover, it has been suggested that the APAIS may be associated with pain levels in the early postoperative period [12].

The purpose of this study was to translate the APAIS into French and to evaluate the psychometric properties of the French version of APAIS.

## Material and methods

### Study population

The sample consisted of French-speaking patients who underwent various procedures, including orthopaedic, hand, plastic, and abdominal surgery, in three university hospitals in southeastern France. Several types of anaesthesia, ranging from regional to general, were represented. Ambulatory procedures were also included. Sociodemographic and other clinical data, such as the American Society of Anaesthesiologists (ASA) physical status measure, which assesses the fitness of patients before surgery, were collected.

The APAIS is a self-report questionnaire comprising six items (see Tables 1 and 2). Two items are dedicated to the assessment of anaesthesia-related anxiety, two items assess surgery-related anxiety, and two items evaluate the desire for information. Thus, the APAIS assesses anxiety about anaesthesia, anxiety about surgery (with the sum of both serving as the global anxiety index), and the desire for information. Patients older than 18, undergoing elective surgery (except obstetric), able to understand and read French, and able to complete a self-report questionnaire were eligible for inclusion in the study.

**Table 1:** Principal component analysis (varimax rotation) of the six-item French APAIS questionnaire

**Items**	**Anxiety about anaesthesia**	**Anxiety about surgery**	**Desire for information**
Q1	.88		
Q2	.82		
Q3		.89	
Q4		.82	
Q5			.88
Q6			.87

**Table 2:** List of the 6 APAIS items

**#**	**French items**	**Original items**
1	Je suis inquiet(ète) à propos de mon anesthésie	*I am worried about the anaesthesia.*
2	Je pense continuellement à mon anesthésie	*The anaesthesia is constantly on my mind.*
3	Je désire savoir tout ce qui est possible à propos de mon anesthésie	*I would like to know as much as possible about the anaesthesia.*
4	Je suis inquiet à propos de mon opération	*I am worried about the procedure.*
5	Je pense continuellement à mon opération	*The procedure is constantly on my mind.*
6	Je désire savoir tout ce qui est possible à propos de mon opération	*I would like to know as much as possible about the procedure.*

The items were answered during a consultation with the anaesthesiologist using a five-point Likert scale ranging from 1 (“not at all”) to 5 (“extremely”). We evaluated the redundancy (inter-item correlation), the response rate (missing data), and the skewness in the distribution of the answers (floor and ceiling effect). A rate of missing data higher than 20% and floor or ceiling effects higher than 15% were considered high.

Within 4 to 48 h after surgery, the patients were asked to complete the “Evaluation du Vécu de l’ANesthésie” questionnaire (EVAN) [9,13], which is a validated, multi-dimensional questionnaire that defines a patient’s reported outcome by assessing the patient’s experience during the perioperative period. This scale captures six dimensions (attention, privacy, information, pain, discomfort, and waiting) and a global satisfaction index. The score for each dimension was obtained by computing the mean of the scores for the items related to that dimension. All of the dimension scores were linearly transformed onto a 0-to-100 scale, where 100 indicates the highest possible level of satisfaction and 0 indicates the lowest. The global satisfaction score was computed as the mean of the dimension scores. The purpose of using the EVAN scores was to use a validated scale from the field of anaesthesia to assess the external validity of the APAIS in the perioperative period.

### Validation process

The validation process included two steps. The first step involved the production of a French version of the APAIS that is semantically equivalent to the original version. In the second step, we evaluated the psychometric properties of the French version, including its internal consistency and reliability, differential item functioning, and external validity.

This study meets the requirements of the Declaration of Tokyo [14], and there was no interference in the physician-patient relationship.

### French translation

A forward-backward translation was performed. A native English bilingual translator produced the first draft from the original version. A French bilingual expert then back-translated the items to cross-validate them.

### Psychometric evaluation

The psychometric evaluation covered four domains: internal validity, differential item functioning, external validity, and acceptability.

### Internal validity

The confirmatory factor analysis used the original APAIS questionnaire [10] as a reference. We sought to determine whether the model generated from the results of the original APAIS fit the data collected in France. For this purpose, a maximum likelihood confirmatory factor analysis was conducted using polychoric covariance [15]. The adequacy of the model was explored using a global index that is responsive to sample size and complexity: the root mean square error of approximation (RMSEA) [16]. We also used an incremental index that is less dependent on the sample size: the comparative fit index (CFI). An RMSEA lower than 0.08 indicates a fair fit, and a CFI higher than 0.9 indicates that the model satisfactorily fits the data.

The unidimensionality was assessed through a Rasch analysis. The Partial Credit Model (PCM), which is an extension of the Rasch model for Likert-type responses, was used [17].

The scalability of each dimension was assessed by examining the pattern of item goodness-of-fit statistics (INFIT), and INFIT values between 0.7 and 1.2 indicate that all of the items on the scale tended to measure the same concept.

The dimensional structure of the questions on the APAIS questionnaire was also explored using a multi-trait, multi-item analysis. Each item was matched with its own dimension, and the item-internal consistency (IIC) was retained if the correlation exceeded the standard of 0.4 after the overlap correction was performed. If an item was more strongly correlated with its dimension than with the other dimensions, we considered this result evidence of the item’s discriminant validity (IDV) [18,19].

The reliability of the internal consistency of each dimension was assessed using the Cronbach’s alpha coefficient. A Cronbach’s alpha coefficient higher than 0.7 was expected for each scale [20].

### Differential item functioning

We evaluated the differential item functioning (DIF) to assess the APAIS’ cross-population properties. DIF analyses were performed to explore the performance of the items and dimensions across several groups of patients (e.g., based on age, gender, type of anaesthesia and surgery, premedication, ASA physical status, and ambulatory course). If an item functioned differently in a subgroup of patients, the DIF would be increased. We calculated the uniform DIF to determine the probability of giving a particular answer at a given level of anxiety or desire for information across the subgroups. The Crane and Larson model [21] was used to detect the DIF, which allowed us to quantify the magnitude of the DIF that signifies an increase in the explained variance of an item by including the variable for each subgroup.

### External validity

We explored the external validity with t-tests by gathering various sets of data, such as age, gender, ASA status score, and EVAN score, to assess the perioperative patient experience.

### Acceptability

The percentage of missing answers was used to explore the global acceptability of the French APAIS among the patients. To ensure data quality, the validation analysis was not performed on records with more than 25% of the responses missing.

## Results

### Study population

A database with 175 patients was created. Women represented 57% of the population, and the mean age was 51 years. Of the patients, 56% had an ASA status score of less than 2 (Table 3). The mean APAIS scores were as follows: anxiety for anaesthesia (3.3 ± 1.8); anxiety for surgery (3.9 ± 2.3); global anxiety (7.2 ± 3.7), and desire for information (5.7 ± 2.3) (Table 4).

**Table 3:** Descriptive characteristics of the sample

		**N = 175 (%)**
Gender	Female	99 (56.6)
	Male	76 (43.4)
ASA score	1	98 (56)
	2	71 (40.6)
	3	3 (1.7)
	4	3 (1.7)
General anaesthesia		44 (25.1)
Regional anaesthesia		131 (74.9)

**Table 4:** Item internal consistency (IIC), item discriminant validity (IDV), percentage of missing values (%MV), Cronbach’s alpha (alpha), intra-class correlation coefficient (ICC) of the APAIS dimension scores and global index (mean +/- SD), and inlier-sensitive fit (INFIT)

**Dimension/APAIS dimensions**	**Mean ± SD**	**Floor**	**Ceiling**	**MV (%)**	**IIC**	**IDV**	**Alpha**	**INFIT**
Anxiety about anaesthesia (2)	3.3 ± 1.8	43.1	1.7	1.7	0.67	0.16-0.61	0.80	0.94-1.09
Anxiety about surgery (2)	3.9 ± 2.3	40.2	2.9	0.6	0.72	0.27-0.61	0.84	0.98-1.01
Desire for information (2)	5.7 ± 2.3	11.5	6.9	2.9	0.61	0.16-0.36	0.76	0.96-1.00
Global anxiety	7.2 ± 3.7	NA	NA	5.1	NA	NA	NA	NA

*IIC* the correlation between the item scores and their dimension score (corrected for overlap). The numbers shown are the lowest and highest Pearson’s correlation coefficients. *IDV* the correlation between the item scores for a given dimension and the other dimension scores. The numbers shown are the lowest and highest Pearson’s correlation coefficients. *NA* not applicable.

### French translation

There were no mismatches in the cross-validation of the items after they were back-translated; see Table 1.

### Psychometric evaluation

The French model had the same structure as the original APAIS and explained 85% of the total variance.

### Internal validity

The principal component factor analysis revealed the same three-dimensional structure as the original scale: anxiety about anaesthesia (two items), anxiety about surgery (two items), and the desire for information (two items). The characteristics of the items and the scales for each dimension are reported in Table 2.

The confirmatory factor analysis indicated a good fit (RMSEA = 0.069 and CFI = 1.00).

Of the items fitted to the Rash model, none produced an INFIT statistic outside the acceptable range, which indicates that the French version of the APAIS is scalable.

The item internal consistency (IIC) ranged from 0.61 to 0.72. The correlations between the items and the other dimensions (i.e., the item discriminant validity (IDV)) ranged from 0.16 to 0.61.

The internal consistency reliability and construct validity were high, i.e., the Cronbach's alpha values ranged from 0.76 to 0.84.

### Differential item functioning

The level of DIF was low between the subgroups of patients based on age, gender, type of anaesthesia or surgery, premedication, ASA physical status, and ambulatory course (Table 5).

**Table 5:** Differential item functioning (DIF) analyses and assessment of unidimensionality by inlier-sensitive fit (INFIT)

**Dimension/APAIS dimensions**	**DIF**	**INFIT**
Anxiety about anaesthesia (2)	0.80	0.94-1.09
Anxiety about surgery (2)	0.84	0.98-1.01
Desire for information (2)	0.76	0.96-1.00
Global anxiety	NA	NA

*IIC* the correlation between the item scores and their dimension score (corrected for overlap). The numbers shown are the lowest and highest Pearson’s correlation coefficients. *IDV* the correlation between the item scores for a given dimension and the other dimension scores. The numbers shown are the lowest and highest Pearson’s correlation coefficients. *NA* not applicable.

### External validity

The French version of the APAIS was compared with other concurrent data to assess its convergent validity (Table 6). Patients older than 55 tended to feel more anxious about anaesthesia. Female patients experienced significantly greater anxiety about anaesthesia and a greater desire for information. There was no difference between the patients who received general anaesthesia compared with the patients who received regional anaesthesia. The APAIS was strongly correlated with the dimensions of the EVAN (Table 7). Anxiety about anaesthesia, global anxiety, and the desire for information were correlated with the dimensions of discomfort, waiting, and pain and with the global index of the EVAN. As expected, because the EVAN aims to evaluate a patient’s experiences with anaesthesia, anxiety about surgery was not correlated with any of the EVAN dimensions except the discomfort dimension.

**Table 6:** Correlations between the Amsterdam perioperative anxiety and information scale (APAIS) scores and age, gender, and type of anaesthesia (T-tests)

**APAIS**	**Anxiety about anaesthesia**	**Anxiety about surgery**	**Global anxiety**	**Desire for information**
Age				
<55 years (97)	3.2 ±1.8	4 ±2.3	7.2 ±3.6	5.6 ±2.2
> = 55 years (78)	3.5 ±1.8	3.8 ±2.3	7.3 ±3.7	5.9 ±2.3
*T* test	0.07	0.39	0.92	0.28
Gender				
Female (99)	3.5 ±1.7	4.1 ±2.4	7.5 ±3.7	6 ±2.3
Male (76)	3.1 ±1.8	3.7 ±2.2	6.8 ±3.6	5.3 ±2.2
*T* test	0.01	0.44	0.08	0.05
Anaesthesia				
General (44)	3.4 ±1.8	4.1 ±2.6	7.5 ±3.8	5.8 ±2.3
Regional (131)	3.3 ±1.8	3.9 ±2.2	7.1 ±3.6	5.7 ±2.3
*T* test	0.6	0.94	0.54	0.88

**Table 7:** Comparisons between the EVAN-LR scores and the Amsterdam perioperative anxiety and information scale scores

**APAIS**	**Anxiety about anaesthesia**	**Anxiety about surgery**	**Global anxiety**	**Desire for information**
Evan-LR				
Attention	-0.05	-0.01	-0.02	0.01
Information	-0.13	-0.08	-0.09	-0.17
Discomfort	-0.32**	-0.23**	-0.27**	-0.19*
Pain	-0.19*	-0.12	-0.22*	-0.21*
Waiting	-0.20*	-0.13	-0.22*	-0.19*
Index	-0.20*	-0.16	-0.21*	-0.19*

*p < 0.05, **p < 0.01. The numbers shown are the Pearson’s correlation coefficients.

### Acceptability

For each dimension, the proportion of missing values was low (ranged from 0.6 to 2.9%). These results indicate that the questionnaire was well accepted.

## Discussion

Patient-reported outcomes are becoming widespread, but there are still questions regarding the discrepancies between the patients’ subjective feelings across cultures and their implications on the measurements of satisfaction and anxiety.

The APAIS has been initially designed and validated in dutch, the construct validity was evaluated by factor analysis with rotation in a population of 320 dutch patients while external validity been performed in 200 patients. Since then, several works have assessed APAIS validity among populations with different languages and cultures highlighting the need for a French version as well. The first adaptation was made by Boker et al. [11] in a population of 197 English speaking Canadian patients. One strength of this study was to compare APAIS with STAI and anxiety visual analogue scale (VAS). Applicability of APAIS was supported with a time of completion of 2 vs 5–7 minutes for the STAI. APAIS correlated well with STAI. A Japanese validation was performed in a population of 126 patients the same year [22] and the last adaptation of APAIS was made in German language among 68 patients in 2007 [23]. The German validation emphasized external validity by comparing APAIS with several scale: the Hospital Anxiety and Depression Scale (HADS); the Self-rated symptom CheckList (SCL-9-K); The COping with Surgical Stress scale (COSS); the KASA scale and the State Operation Anxiety Scale (STOA).

The study of validation of the French version of APAIS had two objectives.

First, we aimed to further support its generalisability. Following international guidelines, we demonstrated the strong psychometric properties of the French version of the APAIS. We explored its external validity by comparing it with an original validated scale for evaluating the entire patient experience during the perioperative period: the EVAN. Anxiety is assumed to be an important determinant of the patient experience during the perioperative period, but studies are still lacking. By gathering EVAN data for the validation of the APAIS, we explored the link between anxiety and the overall patient experience. The strong correlations supported that the French version of the APAIS is correlated with patients’ experiences. In addition to the EVAN, the external validity of the APAIS was also explored by examining the correlation with other concurrent measures, such as gender and age, which supported the convergent validity of the questionnaire.

Second, the growing data about APAIS validity among various populations tends to assume that anxiety is a steady measure among patients. We would like to explore this hypothesis and finally, this study is the first to assess differential item functioning on the APAIS for various subgroups of patients. The fact that there were no observable differences by gender, age, ASA score or type of anaesthesia (regional or general) adds important information to consider when using the APAIS in various clinical practice settings.

Nevertheless, some limitations should be mentioned. The study sample included patients undergoing both regional and general anaesthesia procedures, which might have introduced some unexpected variability. However, to date, there are no data in the literature that indicate a relationship between a patient’s anxiety level and the type of anaesthesia procedure. Another limitation is that we did not compare the APAIS to another anxiety scale like previous study did. We believe that there is enough data in literature to support APAIS value to assess patient’s anxiety in the preoperative period [10,11,22,23]. But the other property, like differential item functioning among patient’s subgroups still had to be demonstrated and was the object of the second objectives of this study.

Besides those metrical aspects, assuming that APAIS is now validated in Dutch, English, Japanese, German and French language, the next step is probably a broader integration of patient anxiety in care process. Early screening of anxious patients could enable specific strategies to improve their experience of the perioperative period [24]. As an example we could imagine basing the premedication strategies upon the APAIS score by giving sedatives just before surgery to the most anxious patients. Tools for a patient oriented healthcare are available from now and making studies with “patient reported outcome” as a main objective represents the next step.

## Conclusions

The French version of the APAIS is valid and reliable. This tool enables the evaluation of anxiety among French patients undergoing anaesthesia. Taking a patient’s experiences into account through the assessment of patient-reported outcomes with measurement tools such as the APAIS is a step toward quality improvement in anaesthesia.

## Competing interests

The authors declare that they have no competing interests.

## Authors’ contributions

AMS has collected the original data of the study, analyzed the data, wrote the final manuscript, and is the author responsible for archiving the study files; AL has seen the original study data, analyzed the data, and approved the final manuscript; XC has seen the original study data, reviewed the analysis of the data and approved the final manuscript; NB has seen the original study data, reviewed the analysis of the data and approved the final manuscript; PA has seen the original study data, reviewed the analysis of the data and approved the final manuscript. All authors read and approved the final manuscript.

## Authors’ information

AP-HM Assistance Publique-Hôpitaux de Marseille, 264 rue Saint Pierre, 13385 MARSEILLE PACA, France.
